# The APRU Global Health Program: Past and Future

**DOI:** 10.2188/jea.JE20160049

**Published:** 2016-04-05

**Authors:** Jonathan Samet, Mellissa Withers

**Affiliations:** University of Southern California Institute for Global Health, Los Angeles, CA USA

**Keywords:** Pacific Rim, Asia-Pacific, education, training, universities, global health

## Abstract

The Association of Pacific Rim Universities (APRU) is an international consortium of 45 universities in the Pacific Rim, representing 16 economies, 130 000 faculty members and more than two million students. The APRU Global Health Program aims to expand existing collaborative research efforts among universities to address regional and global health issues. Since its launch in 2007–08, the program has covered a significant range of topics including emerging public health threats, ageing and chronic diseases, infectious diseases and health security issues, among others. The Program’s activities in research, training, and service around the globe illustrate the diverse dimensions of global health. In this paper, the major activities to date are outlined and future planned activities are discussed.

## INTRODUCTION

This article is based on a speech presented at the 2015 Global Health Workshop of the Association of Pacific Rim Universities (APRU) hosted by Osaka University, on 30–31 October 2015, in Osaka, Japan. The objective is to give an overview of APRU Global Health Program, outlining the previous activities and future plans.

## APRU HISTORY

The Association of Pacific Rim Universities (APRU) is an international organization of leading research universities in the Pacific Rim region. The region extends to much of the world. Within APRU, there are now 45 member universities representing many large and important economies, 140 000 faculty members, and several million students, as shown in Table 1 and Table 2. APRU was established in 1997. The President of the University of Southern California (USC) at the time, Steven Sample, was one of the leaders in suggesting the idea that an organization of Pacific Rim Universities would be valuable. He believed that there would be synergies from the connections among leading research institutions that focus on a broad and common region. The Presidents from three other California-based universities agreed and formed the network. These included: 1. California Institute of Technology (Thomas Everhart); 2. University of California, Berkeley (Chang-Lin Tien); and 3. University of California, Los Angeles (Charles Young).

**Table 1.** APRU at a glance

45 leading research universities
17 APEC economies including the world’s 3 largest
2 million students
Culturally dynamic and diverse

**Table 2.** Member Universities by region

Country/Region	University	Vice-Chancellor and/or President/Principal	APRU Senior Staff Contact
Australia	Australian National University	Brian P. Schmidt	Shirley Leitch
	University of Melbourne	Glyn Davis	Susan Elliott
	University of Sydney	Michael Spence	—
	UNSW Australia	Ian Jacobs	Fiona Docherty
Canada	University of British Columbia	Martha Piper (Interim)	—
Chile	University of Chile	Ennio Vivaldi Véjar	Pia Lombardo
China and Hong Kong SAR	Fudan University	Xu Ningsheng	Zhu Chouwen
	Hong Kong University of Science and Technology	Tony F. Chan	Eden Yi-Teng Woon
	Nanjing University	Chen Jun	Zhang Rong
	Peking University	Lin Jianhua	Yansong Li
	Tsinghua University	Qiu Yong	Jinliang Li
	University of Hong Kong	Peter Mathieson	John Kao
	University of Science and Technology of China	Wan Lijun	Jiajie Jiang
	Zhejiang University	Wu Zhaohui	Li Min
Chinese Taipei	National Taiwan University	Pan-Chyr Yang	Luisa Shu-Ying Chang
Indonesia	University of Indonesia	Muhammad Anis	Melda Kamil Ariadno
Japan	Keio University	Atsushi Seike	Jiro Kokuryo
	Kyoto University	Juichi Yamagiwa	Junichi Mori
	Osaka University	Shojiro Nishio	Toshiya Hoshino
	Tohoku University	Susumu Satomi	Toshiya Ueki
	University of Tokyo	Makoto Gonokami	Ken Furuya
	Waseda University	Kaoru Kamata	Norimasa Morita
Korea	Korea University	Jaeho Yeom	Sunhyuk Kim
	Seoul National Unversity	Nak-in Sung	Seong Ho Sheen
	Yonsei University	Yong-Hak Kim	Joongi Kim
Malaysia	University of Malaya	Mohd Amin Jalaludin	Awang Bulgiba Awang Mahmud
Mexico	Monterrey Institute of Technology	Salvador Alva	Joaquin Guerra Achem
	National Autonomous University of Mexico	Enrique Graue Wiechers	Eduardo Garcia Barzana
New Zealand	University of Auckland	Stuart McCutcheon	Jenny Dixon
Philippines	University of Philippines	Alfredo E. Pascual	Gisela P. Concepcion
Russia	Far Eastern Federal University	Sergey V. Ivanets	Vladimir Kurilov
Singapore	National University of Singapore	Tan Chorh Chuan	Andrew Wee
Thailand	Chulalongkorn University	Pirom Kamol-Ratanakul	Kriengkrai Boonlert-U-Thai
USA	California Institute Technology	Thomas F. Rosenbaum	—
	Stanford University	John Hennessy	—
	University of California, Berkeley	Nicholas Dirks	Laurie Goldman
	University of California, Davis	Linda P.B. Katehi	Joanna Regulska
	University of California, Irvine	Howard Gillman	James Hicks
	University of California, Los Angeles	Gene D. Block	Cindy Fan
	University of California, San Diego	Pradeep K. Khosla	Peter F. Cowhey
	University of California, Santa Barbara	Henry T. Yang	Michael Stohl
	University of Hawai’i at Manoa	Robert Bley-Vroman	R. Anderson Sutton
	University of Oregon	Michael H. Schill	Dennis Galvan
	University of Southern California	C.L. Max Nikias	Shantanu Dutta
	University of Washington	Ana Mari Cauce	Jeffrey Riedinger

As of 2016, the APRU headquarters will be located in Hong Kong. Dr. Christopher Tremewan is the current Secretary-General. APRU has many activities and programs, of which the Global Health Program is one. Other programs are: multi-hazards, which focuses on the different threats to the well-being and economies of the countries in the regions; sustainable cities; and population and aging, clearly very important topics for all member countries. There is an annual meeting of the Presidents of APRU member universities, most recently held in June 2015 at Osaka University. The President of USC, Max Nikias, is currently the chair of APRU.

## APRU GLOBAL HEALTH PROGRAM HISTORY

The countries that form the Pacific Rim, despite language differences, cultural differences, and economic differences, are linked in many ways. What happens in this region is an important driver of global health. The Global Health Program began as the APRU World Initiative (AWI), but the name “Global Health Program” seemed more appropriate and as a result the name was changed several years ago. Launched in 2007/2008, the Program has addressed a wide range of topics including non-communicable diseases and the rising epidemics that threaten our region in terms of infectious diseases. Whenever a moment of calm during which infectious disease control is reached, a new disease seems to emerge. Examples include AIDS in the early 1980s and SARS in 2003. In the United States, West Nile Virus is now considered as an endemic virus, and Chikungunya has arrived, making its way from Asia to the United States and spreading into Europe. Unfortunately, many public health concerns have become globalized.

The Program is housed within the University of Southern California. Dr. Mellissa Withers is the Program leader and sits within the Institute for Global Health at the USC. Also at USC, Dr. Jonathan Samet is the chair of the Global Health Program. An Advisory Group of 12 additional members from ten economies provide leadership, as shown in Table 3.

**Table 3.** APRU GHP Advisory Group Members for 2014–2016

Name	University
Chang-Chuan Chan	National Taiwan University
Sun Ha Jee	Yonsei University
Masamine Jimba	University of Tokyo
Jamal Hisham	United Nations University
Judith McCool	University of Auckland
Jonathan Samet	(Chair) University of Southern California
Marc Schenker	University of California, Davis
Terry L. Schmidt	University of California, Irvine
Giorgio Solimano	University of Chile
Richard Taylor	University of New South Wales
Heather Wipfli	University of Southern California
Bambang Wispriyono	University of Indonesia
Mellissa Withers	(Program Manager) University of Southern California
Shankuan Zhu	Zhejiang University

The focus of the Program has been on education, a topic common to all members, and research. However, the members also have a shared interest in policy. Trying to make certain that the research that is carried out will make a difference is an important goal. Most members are in global health because they hope the work they do impact policy and lead to improvements in global health. They face the common challenge of how to affect policy—how do you go from a paper in a nice epidemiological public health journal, to making something happen? Perhaps the power of the APRU can be harnessed to facilitate such translation. The Program was recently cited in a paper by Liu et al. as a good example of global health collaboration. This paper comments on the existence of APRU, and discusses APRU’s interest in creating global health leadership.^1^

## THE ANNUAL WORKSHOP

This is a highly interdisciplinary group, demonstrated nicely by this year’s workshop program. There are many faculty present but also an increasing number of students. A recent change in the workshop, begun last year at National Taiwan University, was the very important idea of having more students present posters and participate. The workshops are opportunities for the next generation or two to come together and meet people from APRU Universities.

Where was the first meeting and when was it held? Table 4 shows where the annual workshops have been held. The 10th anniversary meeting will be held in 2016 in Sydney, Australia and will be hosted by the University of New South Wales. The 2014 workshop was held in Taiwan at National Taiwan University. Two hundred forty participants attended from 18 economies and 17 distinct disciplines. And at the 2015 workshop, the number of participants has reached a record level. Nineteen economies and 14 disciplines are represented at this workshop, as seen in Table 5.

**Table 4.** Annual APRU Global Health Workshops

Year	Location	Date
2007	Peking University	May 24–26
2008	University of Tokyo	June 23–25
2009	Johns Hopkins University	June 24–26
2010	Nanjing University	June 17–19
2011	University of Indonesia	June 13–16
2012	University of Southern California	June 23–26
2013	Zhejiang University	October 29–November 1
2014	National Taiwan University	September 24–27
2015	Osaka University	October 30–November 1
2016	UNSW Australia	September 28–30

**Table 5.** 2015 workshop participants

APRU Members	Non-APRU Members
Chulalongkorn University	Baika Women’s University
Far Eastern Federal University	California State University Northridge
Fudan University	Cavite State University
Keio University	Chiba University
Korea University	City University of Marikina
Kyoto University	Duke University
Monterrey Institute of Technology	Duke Kunshan University
National University of Singapore	Fooyin University
Osaka University	Fukushima Medical University
Peking University	Hanoi School of Public Health
Seoul National University	Hassanuddin University
National Taiwan University	Kainan University
Tohoku University	Mahidol University
University of Auckland	Mie Graduate School of Medicine
University of California, Irvine	Morinomiya University of Medical Sciences
University of California, Los Angeles	Mukogawa Women’s University
University of Indonesia	Nagoya University School of Medicine
University of Malaya	Nara Women’s University
University of New South Wales	National University of Malaysia
University of Philippines	Osaka Medical College
University of Southern California	Saga University
University of Sydney	Shiga University of Medical Science
University of Tokyo	Sookmyung Women’s University
	Syarif Hidayatullah State Islamic University
	United Nations University
	University of Ghana
	University of the East Ramon
	University of Manchester
	University of Tsukuba
	Warmadewa University

Many people have helped guide the development of the annual Global Health Program meetings. There’s a group of people who remain interested in planning the activities and we welcome more, particularly these interested, in meeting at odd hours by Skype. If one person is having a 9 am conference call, somebody else is having an 11 pm conference call, or sometimes a 1 am call. Despite such challenges, a core group has worked together in planning these meetings.

## PROGRAM ACTIVITIES

Below other major activities of the APRU Global Health Program are highlighted.

**WORKING GROUPS:** Five working groups have been formed around key issues of interest to members. The topics are: 1. Environmental Health; 2. Global Health Education and Technology; 3. Nursing; 4. Non-Communicable Diseases; 5. Trade, Security and Migration. The objectives of these groups are to create collaborations, share information, and develop projects. Small-group discussions within these groups are held regularly regarding global health issues. The Groups are encouraged to undertake projects, such as writing papers, hosting webinars, and conducting research.

**STUDENT POSTER CONTEST:** The first APRU Global Health Program student poster contest was held in 2014. This year we had 40 entries and three winners in the undergraduate and graduate categories were chosen. Winners exhibit their posters at the annual workshop and receive certificates and monetary awards. This is a good example of an educational program that reaches across the Pacific Rim.

**DISTANCE EDUCATION COURSE:** Another exciting venture is currently under way, led by Dr. Terry Schmidt at the University of California, Irvine and Mellissa Withers at USC. This online course, called Global Health LIVE! is a graduate seminar for Master of Public Health students. The course extends the theory of global health to the practice of global health. The relationship of health, foreign policy, and global health leadership are the scope of the course. Participants from five universities are involved, including University of Southern California, University of California at Irvine, National Taiwan University, University of Tokyo and Chinese University of Hong Kong. A live seminar was held once per week for two hours via live video. Students attended the live session simultaneously for 10 weeks. An international guest lecturer and global health leader spoke during each session. Then students interacted with the lecturer and with each other. One of the major assignments of the course was to work in groups (from the four different universities) to create a video on a global health leadership topic. Guest lecturers included world-renowned global health leaders, including Greg Martin, Larry Gostin, Bill Magee, Al Sommer, and others. The main learning outcomes of the course were: 1. Understand key current issues in global health; 2. Expand networking opportunities for future global health career opportunities; 3. Practice and hone presentation skills and participate in case studies; 4. Construct a Mission, Vision, and Values (Personal) Career Statement and 5. Describe global health leaders’ characteristics. The complexities of offering a multi-university course presented many challenges. However, these were resolved and the course received very impressive student evaluations. This course and other similar courses will be offered again in the future.

**GRANTS:** One goal of the Program is to bring together expertise and develop new projects. There have been efforts to seek grant funding to support APRU activities. With APRU colleagues, a grant was submitted to the Fogarty International Center of the NIH for training in Bioethics. Tobacco control is also a key issue for everyone in the region. Members from USC and Fudan University put together a plan for a coalition to work for tobacco control in China. And Fudan was successfully funded by Bloomberg philanthropies to develop and implement a tobacco control advocacy and leadership program.

**COLLABORATIVE SCHOLARLY PAPERS:** Several manuscripts have come out from the work at these meetings. The first one, titled, “Training the next generation of global health experts: experiences and recommendations from Pacific Rim universities” relates to education and training and will be published in the journal *Globalization and Health*. Authors from multiple countries participated, including USC, the University of Tokyo, the University of Auckland and National Taiwan University.

Several other manuscripts are under development. One provides a commentary on how some of the region’s countries have approached setting air quality standards. This manuscript provides useful lessons learned from multiple countries. The authors are from USC, National Taiwan University, University of Auckland, National University of Singapore, National University of Malaysia, Fudan University and Seoul National University. Another example is a paper that was developed from a special workshop on global health competencies at the 2014 annual meeting. Titled “Global Health Leadership: Core Competencies in Graduate-level Global Health Education”, the authors are members of the Global Health Education and Technology Working Group from USC, National Taiwan University, University of California at Irvine and University of the Philippines.

**TOBACCO SURVEY:** In the United States and some other countries, electronic cigarettes are a rising threat. The most recent device is a very sophisticated, essentially electronic drug delivery, system. Those who use them are able to mix their own solution, so called e-juice, and add the flavorings they want. They can also vary the nicotine concentration. University students are a key risk group for becoming e-cigarette users and becoming exposed and addicted to nicotine. An online survey of students at APRU member universities will be disseminated next year. The survey will collect data on students’ views of tobacco control and familiarity with electronic cigarettes.

**NEWSLETTER:** A quarterly newsletter is published covering different aspects of what the APRU Global Health Program institutions and members are doing.

## LOOKING FORWARD

In terms of the future, members have proposed a number of projects. A priority is the shared interest that members have in shaping policy in the region. One thought has been that the APRU university Presidents might come together to make statements on important issues for the region. Since USC President Max Nikias currently leads APRU, timing is optimal to discuss such possibilities.

Members have also expressed strong interest in conducting more multi-country studies, one of the most powerful opportunities for APRU members. The biggest barrier, and one that needs more attention, is to identify funding agencies that will support such multi-country studies. The Program presents a unique way to work with students, local communities, and governments, to carry out important research projects, but funding for these projects unfortunately tends to be more nationally-oriented. Over the past several years, many different funding agencies have been approached but these studies do not seem to fit within the usual idea of what the funding should look like.

Members have also expressed the desire for more training, curriculum development, and distance education. Substantial initial effort was made and the lessons learned from this original distance education course provided many lessons to be learned. Two more distance education courses are being planned for 2016.

Another suggestion was to create special journal editions for APRU papers, a prospect realized with this meeting. This issue and possible future issues will sustain the visibility of the Global Health Program.

One new activity planned for 2016 is the APRU Global Health Case Competition. Such competitions have worked well in the United States. It involves groups of students addressing important global health problems in sort of a debate situation. At USC we had internal competition as other universities to go to a national competition at Emory University. This activity has become very popular, attracting groups from within the United States, but also from outside the United States including Mexico.

## CONCLUSIONS

How can you become more involved? Mellissa Withers is the focal point for activities; interested parties can contact her with ideas and interests. They can also join the listserv and visit the Facebook page. Members are encouraged to send news items to be included in the quarterly newsletter. Universities can also help disseminate the newsletter. Even more possibilities to get involved exist. Think about research projects that could be done uniquely through the APRU mechanism. Anyone who has ideas about where to seek funding for these projects is also welcome to contact us.

We have an opportunity over the next few days to make what is becoming an increasingly effective mechanism even more effective. I look forward to speaking with everyone. This is a key opportunity for us to network and learn about each other.

## ONLINE ONLY MATERIAL

eFigure 1. Group picture of the 2015 Global Health Workshop of APRU attendees.
