# Supplementary figures and images for: The APRU Global Health Program: Past and Future
Source: J Epidemiol. 2016 Apr 5;26(4):166–70. doi: 10.2188/jea.JE20160049 (PMC4808682; doi:10.2188/jea.JE20160049)

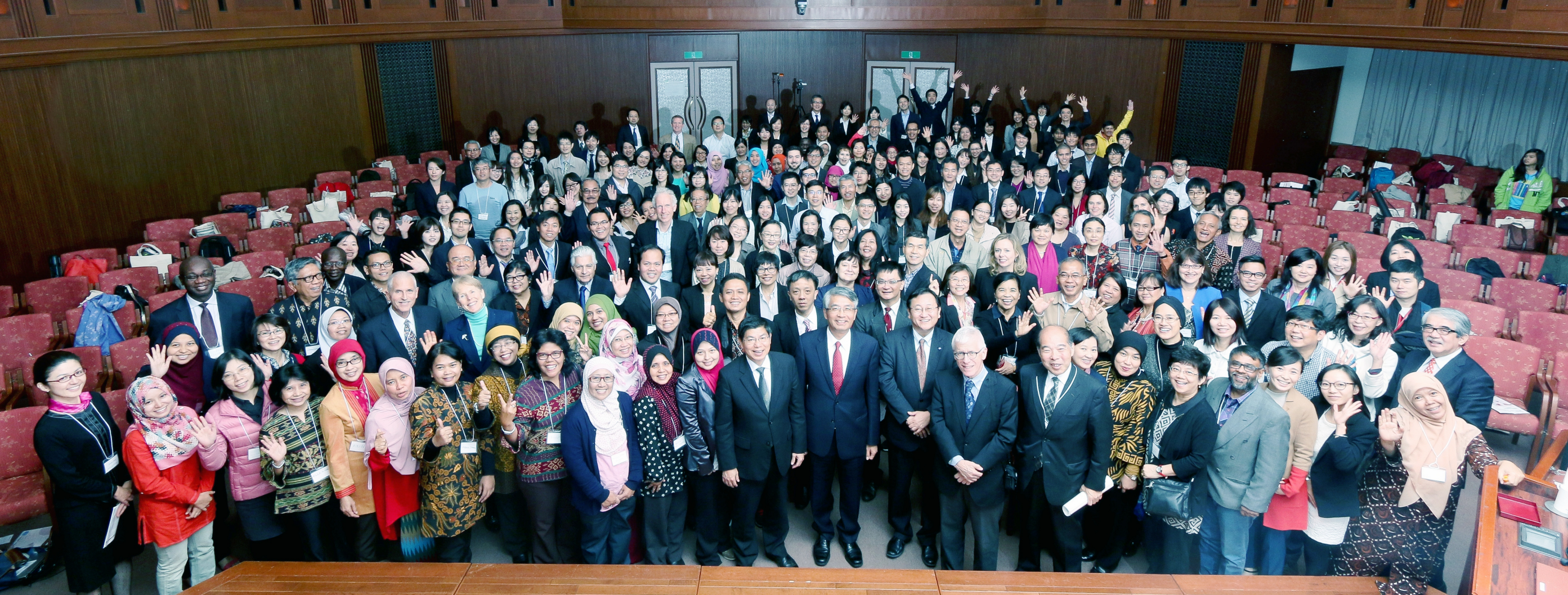

Supplement: eFigure 1. [file je-26-166-s001.pdf]
